# Supplementary material for: ProteinShader: illustrative rendering of macromolecules
Source: BMC Struct Biol. 2009 Mar 30;9:19. doi: 10.1186/1472-6807-9-19 (PMC2672931; doi:10.1186/1472-6807-9-19)
Supplement: Additional file 1 — ProteinShader program without source code. This compressed file contains the complete ProteinShader program including associated libraries, but no source code. A README.txt file gives an overview of the ProteinShader distribution, and the index.html file in the help subdirectory has directions on getting started with the program as well as a set of tutorials. [file 1472-6807-9-19-S1.zip › ProteinShader-beta-0_9_4-binary/help/api/org/proteinshader/gui/viewing/Rotation.html]

Rotation (ProteinShader API)


|  |  |  |
| --- | --- | --- |
| **Overview** \| **Package** \| **Class** \| **Use** \| **Tree** \| **Deprecated** \| **Index** \| **Help** | |  |
| **PREV CLASS**   **NEXT CLASS** | **FRAMES**    **NO FRAMES**     **All Classes** |  |
| SUMMARY: NESTED \| FIELD \| CONSTR \| METHOD | DETAIL: FIELD \| CONSTR \| METHOD |  |


---


## org.proteinshader.gui.viewing Class Rotation

```
java.lang.Object
  org.proteinshader.gui.viewing.Rotation
```

---

``` public class Rotation extends Object ```

Keeps track of rotation transformations of the current model.

---

| **Field Summary** | |
| --- | --- |
| `static double` | `HALF_PI`             One-half Pi is defined as 1.57079632679489, and is equal to 90 degrees. |
| `static double` | `PI`             Pi is defined as 3.14159265358979, and is equal to 180 degrees. |
| `static double` | `RADIANS_PER_DEGREE`             Pi divided by 180 degrees. |
| `static double` | `TWO_PI`             2\*Pi is defined as 6.28318530717958, and is equal to 360 degrees. |
| `static Vec3d` | `X_AXIS`             Vector representing an x-axis. |
| `static Vec3d` | `Y_AXIS`             Vector representing an y-axis. |
| `static Vec3d` | `Z_AXIS`             Vector representing an z-axis. |


**Constructor Summary**

`Rotation(Mediator mediator,
FpsLabel fpsLabel)`
  
          Constructs a Renderer.


| **Method Summary** | |
| --- | --- |
| `void` | `calculateTimeAndRotation()`             Calculates an x-axis and y-axis rotation based on the current system time and the previous time this method was called. |
| `double` | `getScale()`             Returns the scale factor for the image. |
| `void` | `loadFpsFontFile(GL gl)`             Tells the FpsLabel object to read in the font file that it needs for displaying a frames per second label on the canvas. |
| `void` | `rotateAboutXY(double deltaX, double deltaY)`             Rotates the model about the x-axis and y-axis. |
| `void` | `rotateAboutZ(double deltaZ)`             Rotates the model about the z-axis. |
| `void` | `rotateScaleAndCenterModel(GL gl)`             This helper method for display() rotates, scales, and centers the model. |
| `void` | `setCenter(double x, double y, double z)`             Sets the xyz-coordinates for the center of gravity of the current model. |
| `void` | `setImageOrientation(OrientationEnum orientation)`             Resets the orientation of the image by resetting the angle of rotation about the x-axis and the angle of rotation about the y-axis. |
| `void` | `setScale(double scale)`             Sets the scale factor for the image. |
| `void` | `startAnimation(double xAxisSpeed, double yAxisSpeed, GLCanvas canvas)`             Uses an Animator object to rotate the protein image about its x-axis and/or y-axis. |
| `void` | `stopAnimation()`             Stops the rotation animation that is started with startAnimation(). |
| `void` | `writeFps(GL gl, GLAutoDrawable canvas)`             Updates the frames per second display on the canvas. |

**Methods inherited from class java.lang.Object**

`clone, equals, finalize, getClass, hashCode, notify, notifyAll, toString, wait, wait, wait`

**Field Detail**

### HALF\_PI

```
public static final double HALF_PI
```

:   One-half Pi is defined as 1.57079632679489,
    and is equal to 90 degrees.

    **See Also:**: Constant Field Values

---


### PI

```
public static final double PI
```

:   Pi is defined as 3.14159265358979,
    and is equal to 180 degrees.

    **See Also:**: Constant Field Values

---


### TWO\_PI

```
public static final double TWO_PI
```

:   2\*Pi is defined as 6.28318530717958,
    and is equal to 360 degrees.

    **See Also:**: Constant Field Values

---


### RADIANS\_PER\_DEGREE

```
public static final double RADIANS_PER_DEGREE
```

:   Pi divided by 180 degrees.

    **See Also:**: Constant Field Values

---


### X\_AXIS

```
public static final Vec3d X_AXIS
```

:   Vector representing an x-axis.

---


### Y\_AXIS

```
public static final Vec3d Y_AXIS
```

:   Vector representing an y-axis.

---


### Z\_AXIS

```
public static final Vec3d Z_AXIS
```

:   Vector representing an z-axis.


**Constructor Detail**

### Rotation

```
public Rotation(Mediator mediator,
                FpsLabel fpsLabel)
```

:   Constructs a Renderer.

    **Parameters:**: `mediator` - the Mediator that holds the currently selected model.: `fpsLabel` - the FpsLabel object for writing fps on canvas.


**Method Detail**

### setCenter

```
public void setCenter(double x,
                      double y,
                      double z)
```

:   Sets the xyz-coordinates for the center of gravity of the current model.

    :   **Parameters:**: `x` - the x-coordinate.: `y` - the y-coordinate.: `z` - the z-coordinate.

---


### setImageOrientation

```
public void setImageOrientation(OrientationEnum orientation)
```

:   Resets the orientation of the image by resetting the angle of rotation about
    the x-axis and the angle of rotation about the y-axis.

---


### rotateScaleAndCenterModel

```
public void rotateScaleAndCenterModel(GL gl)
```

:   This helper method for display() rotates, scales, and centers the model.

---


### rotateAboutXY

```
public void rotateAboutXY(double deltaX,
                          double deltaY)
```

:   Rotates the model about the x-axis and y-axis.

    :   **Parameters:**: `deltaX` - the change in angle of rotation about the x-axis.: `deltaY` - the change in angle of rotation about the y-axis.

---


### rotateAboutZ

```
public void rotateAboutZ(double deltaZ)
```

:   Rotates the model about the z-axis.

    :   **Parameters:**: `deltaZ` - the change in angle of rotation about the z-axis.

---


### startAnimation

```
public void startAnimation(double xAxisSpeed,
                           double yAxisSpeed,
                           GLCanvas canvas)
```

:   Uses an Animator object to rotate the protein image about its
    x-axis and/or y-axis.

    :   **Parameters:**: `xAxisSpeed` - the x-axis speed in degrees per second.: `yAxisSpeed` - the y-axis speed in degrees per second.: `canvas` - the GLCanvas to create an Animator for.

---


### stopAnimation

```
public void stopAnimation()
```

:   Stops the rotation animation that is started with
    startAnimation().

---


### calculateTimeAndRotation

```
public void calculateTimeAndRotation()
```

:   Calculates an x-axis and y-axis rotation based on the current
    system time and the previous time this method was called. This
    method should only be called after checking that the rotation
    animation flag, m\_animate, is true.

---


### loadFpsFontFile

```
public void loadFpsFontFile(GL gl)
                     throws glFont2.FontReaderException
```

:   Tells the FpsLabel object to read in the font file that it needs for
    displaying a frames per second label on the canvas. If the font file
    cannot be loaded, the FpsLabel attribute will be set to null.

    :   **Parameters:**: `gl` - the current GL object. **Throws:**: `glFont2.FontReaderException`

---


### writeFps

```
public void writeFps(GL gl,
                     GLAutoDrawable canvas)
```

:   Updates the frames per second display on the canvas.

    :   **Parameters:**: `gl` - the current GL object.: `canvas` - the canvas to draw on.

---


### getScale

```
public double getScale()
```

:   Returns the scale factor for the image.

    :   **Returns:**: The scale factor as a double.

---


### setScale

```
public void setScale(double scale)
```

:   Sets the scale factor for the image.

    :   **Parameters:**: `scale` - the scale factor as a double.


---


|  |  |  |
| --- | --- | --- |
| **Overview** \| **Package** \| **Class** \| **Use** \| **Tree** \| **Deprecated** \| **Index** \| **Help** | |  |
| **PREV CLASS**   **NEXT CLASS** | **FRAMES**    **NO FRAMES**     **All Classes** |  |
| SUMMARY: NESTED \| FIELD \| CONSTR \| METHOD | DETAIL: FIELD \| CONSTR \| METHOD |  |


---

# *Copyright © 2007-2008*
